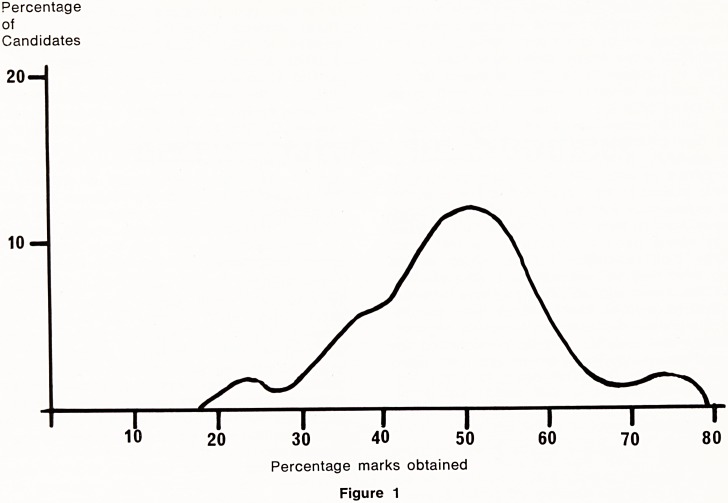# Examinations Using Multiple Choice Questions

**Published:** 1971-01

**Authors:** R. St. J. Buxton

**Affiliations:** Pre-Clinical Dean, The Medica School, University of Bristol


### Examinations Using Multiple Choice Questions

**R. St. J. Buxton, M.B., Ph.D., D.C.H., C.St.J.**

Pre-Clinical Dean, The Medica School, University of Bristol

For many years examinations in the medical course have been of the essay type, but recently there has been a move in the direction of multiple choice questions. In consequence, it has been asked whether this represents progress or if it is merely change for the sake of change. Since the form of the examination requiring the candidate to answer by means of an essay or short notes is well known, but the variety of different question types using multiple choice is less well appreciated, it seems appropriate before making any comparisons to discuss these.

**Multiple Choice questions.**

A. The simplest style is merely to present a statement, and ask the candidate to say whether it is true or false.

Q.1. The normal adult pulse rate at rest tends to be about 72 per minute.

Answer: **True**
False

B. The form of this question can be extended so that the candidate has to make a choice from a number of propositions which are given to him, and usually five are offered.

Q.2. The normal adult pulse rate at rest tends to be about:—

(a) 36
(b) 48
(c) 62
(d) **72**
(e) 105

Both these types are known as Item Assertion or Completion. They are undoubtedly the easiest types to set, but there is a tendency to include in the second form the choices 'All of these' or 'None of these' as for example:—

Q.3. The signs of hyperthyroidism may include:—

(a) Tachycardia
(b) Exophthalmos
(c) Loss of weight
(d) **All of the above** (a, b and c)
(e) None of the above (a, b and c)

The introduction of (d) and (e) reduces the amount of thought a candidate needs to put into his answer, since he will really have only three choices to consider; (e) has been shown by experience to discourage effort and candidates tend to choose this without further thought; so that these are undesirable choices which should be used minimally.

It is undesirable to present questions in a way which necessitates negative thinking since some candidates find this approach particularly difficult and in consequence are discriminated against when this question type is used.

Q.4. is a question of this type:—

Q.4. Active immunisation is available against all of the following diseases EXCEPT:—

(a) Poliomyelitis
(b) Yellow Fever
(c) Rabies
(d) Paratyphoid A
(e) **Malaria**

C. There are two forms of questions called Related Phrase or Association. In the simplest of these a statement or phrase is presented and followed by a second phrase. The candidate is expected to select those choices where the two statements are correctly associated.

Q.5. Which of the following pairs are correct associations?—

| | | |
|---|---|---|
| (a) | Coarctation of the aorta | Positive Wasserman Reaction |
| (b) | Patent ductus arteriosus | Hypertension in arms with hypotension in legs |
| (c) | **Fallot's tetralogy** | **Blue baby syndrome** |
| (d) | Atrial septal defect | Continuous precordial murmur |
| (e) | **Subacute bacterial endocarditis** | **Splinter haemorrhages in skin.** |

This type of question may easily be set in a different way.

(a) Coarctation of the aorta.
(b) Patent ductus arteriosus
(c) Fallot's tetralogy
(d) Atrial septal defect
(e) Subacute bacterial endocarditis.

Q.6. Which of these has hypertension in the arms and hypotension in the legs?

Answer: **a.**

Q.7. Which of these has a continuous precordial murmur?

Answer: **b**

Q.8. Which of these is the most common type of congenital cyanotic heart disease?

Answer: **c.**

D. A third type of question is termed Hypothesis and Evidence. There are various forms of this, but all are based on an assertion being made and a reason being given. The candidate has to decide if the assertion and the reason are correct, and whether the reason correctly explains the assertion.

The candidate is instructed to choose:

(a) if he considers the assertion and reason are true, and the reason correctly explains the assertion.

(b) if he considers the assertion and reason are true but the reason does not explain the assertion.

(c) if he considers the assertion is true but the reason is false.

(d) if he considers the assertion is false but the reason is a true statement.

(e) if he considers both assertion and reason are both false statements.

Q.9. Assertion: Heat is lost from the body by conduction in cold water and cold air at approximately the same rate because
Reason: the limiting factor at low temperatures is the rate at which heat is conducted to the skin from the body core

Answer: Assertion true
Reason true
Explanation true
Therefore correct choice is **(a)**

There is no doubt that this type of question is more suitable for examinations in some subjects than others, but it is also true that the technique of setting them may have to be acquired by practice.

This list of question types is by no means complete but it does give an idea of the different ways information can be extracted from candidates. In medicine it may be acceptable to present a case history and the clinical examination as the background for a series of questions, or the biochemical data may be given so that calculations may be made by the candidate. In fact there are many modifications which are possible and acceptable, some of which will be mentioned in the course of this paper.

**Advantages and Disadvantages of M.C.Q. examining.**

The essay type question is easy to set, but there can always be argument about the marking. It is often said that there is little difficulty in choosing the very poor or the first class student by this method but that the consistency of one examiner and the views of several assessors over a border line candidate are not as uniform as one would wish. Furthermore a large number of scripts to mark with a limited time available provides a heavy load which can be spread only to a limited extent because uniform standards are important and these are most easily achieved with fewer examiners.

The multiple choice question type of examination can examine a subject across a much wider range of topics than the essay type can cover in the same time. The big problem is the setting of questions but this can be done at leisure during the year. Once a bank of questions has been prepared this can be drawn on as required. It is in fact desirable to use questions which have been tried before, since their characteristics and the student response are already known. With a new question it is advisable that it is tried out on colleagues or at least discussed with them if a trial run on a group of students is not possible. Ambiguities in wording can be removed in this way, and in particular the suitability of distractors, that is the wrong choices, can be assessed.

Once the questions are set, the examiners can forget the papers prepared in this way. Candidates can answer the questions by direct marking on the computer cards, and these are machine-punched using an appropriate senser. Results can be assessed using as simple or as complicated a computer programme as one wishes, but the great advantage for the examiner is that he receives the mark list quickly without having to do any marking himself.

**Pass Mark**

When marking MCQ, it is necessary to take into account any incorrect choices which a candidate has made otherwise he will mark all the choices and so get all the correct answers right. There are various ways of making allowance for incorrect choices by deduction of marks but the method accepted should be one which encourages candidates to attempt questions where they are not absolutely sure of the correct answer but are able to make a conditioned guess.

The pass mark in essay type examinations is set by personal experience, and it is customary in many places to use a close marking system based on a fifty per cent pass level. It is not the question difficulty but the marking which determines the pass rate. But when the marking is computerised, only a fraction of the number of correct answers will be recorded, and it is not possible to forecast the student response by varying the question difficulty. It is therefore necessary either to accept a pass mark which will differ with each examination, or to decide on the pass level

**Figure 1**

and then adjust the marks accordingly to a fifty per cent pass mark. Some of the techniques which are used for deciding the pass level are discussed below.

A frequency distribution curve of the marks is presented with the results, and Figure 1 shows the form which this normally takes. The small hump at the upper end of the scale depends on question difficulty in relation to student knowledge and is not always present. The candidates whose results fall on this part of the curve are not necessarily suitable as honours students, since the examination is measuring a different quality of performance from that required of potential honours students.

The position of the main curve on the scale depends on question difficulty. Since the mean and the median differ little in most examinations the curve is symmetrical, and as the scatter of observations is usually considerable, the curve is broad rather than peaked.

There is always a small hump below this which represents the attainment of the weaker students. Changes in question difficulty do not dispose of this curve, which contains the results of those who have not performed satisfactorily.

The pass mark should be specifically selected for each examination. Methods which are used include: all marks below the mean, which gives a high failure rate: or the mean less one (or sometimes two) standard deviations is used as the pass mark: all candidates whose results contribute to the lower hump on the distribution curve are considered to have failed, and then appropriately adjusted for the standard pass (say 50 per cent).

### Additional Information

Because the questions in multiple choice examinations can cover a very wide range of topics, it is less easy for a candidate to have large but undetected gaps in his knowledge. It is also possible to phrase questions to see how complete his understanding is, and to probe his depth as well as breadth of knowledge. The questions may include pictures, either on the question sheet or projected on a screen, so that tissue sections, clinical situations such as a mongoloid face or an electrocardiographic record may be included.

The mark list will show whether a question has not been attempted and the mean and median marks are shown with the range and ranking order of the candidates. This information can be obtained from any examination but because its preparation is tedious the exercise is rarely performed, but with computer marking it is presented as a normal part of the examination record.

When looking at the record of the student reaction to a question, it is useful to see whether the question was too easy (that is if every choice in a question has been correctly marked by every student). At the same time there is no point in making a question so difficult, that few, if any, students get it right. The ideal question should be clearly stated with care to avoid ambiguity so that only the better students are found to have selected the correct and only the correct answers. Two criteria may be used to assess the value of questions asked in an examination. The first is the facilitation index which shows how easy candidates found a question to be. This is based on the number of correct answers selected in each question. The second criterion is known as the discrimination index, which shows how good a particular question is at distinguishing the knowledgeable from the less able students. It is based on a comparison of the performance in each choice of each question of the students placed in the upper half of the class for the whole examination with that of the students placed in the lower half. It does not require a very difficult question to obtain good discrimination, in fact questions having high facilitation indices, which means reasonable ease tend to have excellent discrimination powers. If the lower half of the class have a better showing in any question than the top half, the discrimination index for that question becomes negative. This indicates the question should be checked for wording and phraseology, or perhaps not used again at least in that form.

**Examination Structure.**

The advantages of multiple choice questions have been briefly mentioned above, but the points in favour of essay type questions have not been discussed. These answers show the candidate's ability to write English, to reason and make a critical appraisal of a piece of work, but when the marking of essays is looked at in detail, it is found that most marks are given for the factual material presented which it may be considered can be better assessed by other techniques. On the other hand the assessment of knowledge by multiple choice questions alone does mean that all questions are equally weighted and no indication of the relative importance of different areas of the subject is obtained. Furthermore no discussions or deductions from data are possible. It seems wise therefore that an examination should consist of two or three sections, and the time allocated and the marks apportioned to each section, stated.

The multiple choice section could be accompanied by questions which require answers in the form of short notes, and secondly an essay. There is at present no reason to suppose that the order in which the sections are attempted is important. It has been asked how the marks in the multiple choice and essay questions correlate, and this is being examined in some detail. A small study on limited material suggests that the relationship is linear and that the correlation coefficient is significant at least in the subjects studied.

## Figures and Tables

**Figure 1 f1:**